# Transposon-Based CAR T Cells in Acute Leukemias: Where Are We Going?

**DOI:** 10.3390/cells9061337

**Published:** 2020-05-27

**Authors:** Chiara F. Magnani, Sarah Tettamanti, Gaia Alberti, Ilaria Pisani, Andrea Biondi, Marta Serafini, Giuseppe Gaipa

**Affiliations:** Centro Ricerca M. Tettamanti, Clinica Pediatrica, Università di Milano-Bicocca, Ospedale San Gerardo, 20900 Monza, Italy; cfmagnani@gmail.com (C.F.M.); sarahtettamanti5@gmail.com (S.T.); gaialberti1@gmail.com (G.A.); ilaria.pisani08@gmail.com (I.P.); serafinim72@gmail.com (M.S.); g.gaipa@asst-monza.it (G.G.)

**Keywords:** CAR T-cells, sleeping beauty, transposon, acute leukemia, gene transfer, immunotherapy

## Abstract

Chimeric Antigen Receptor (CAR) T-cell therapy has become a new therapeutic reality for refractory and relapsed leukemia patients and is also emerging as a potential therapeutic option in solid tumors. Viral vector-based CAR T-cells initially drove these successful efforts; however, high costs and cumbersome manufacturing processes have limited the widespread clinical implementation of CAR T-cell therapy. Here we will discuss the state of the art of the transposon-based gene transfer and its application in CAR T immunotherapy, specifically focusing on the Sleeping Beauty (SB) transposon system, as a valid cost-effective and safe option as compared to the viral vector-based systems. A general overview of SB transposon system applications will be provided, with an update of major developments, current clinical trials achievements and future perspectives exploiting SB for CAR T-cell engineering. After the first clinical successes achieved in the context of B-cell neoplasms, we are now facing a new era and it is paramount to advance gene transfer technology to fully exploit the potential of CAR T-cells towards next-generation immunotherapy.

## 1. Background on CAR T-Cell Therapy in Acute Leukemias: Gene Transfer Strategies, Clinical Settings and Accessibility

The concept that in allogeneic hematopoietic transplantation (allo-HSCT) the immune system plays a relevant role in the control of leukemic disease is supported by the clinical observation that immunological effector mechanisms are partly responsible for the elimination of leukemic blasts. Indeed, it is well known that the efficacy of allo-HSCT depends on the anti-leukemic alloreactivity of the donor-derived T-cells, defined as the graft-versus-leukemia (GvL) effect. Moreover, a similar effect is also observed after delayed infusions of donor T-cells. Unfortunately, the beneficial GvL effect occurs concomitantly with an adverse effect associated with allogeneic T-cell activity in normal tissues, called graft-versus-host-disease (GvHD) [1]. Immunotherapy with Chimeric Antigen Receptor (CAR) T-cells arose as a strategy to intensify the GvL and simultaneously reduce the GvHD by exploiting T-cell engineering to provide specificity against a target antigen of interest, which is ideally present only on malignant cells and not on normal tissues [2]. Specifically, CARs are artificial fusion constructs that incorporate an extracellular antigen recognition domain, a transmembrane domain and an intracellular domain including costimulatory and signaling components. The sophistication of the CAR design has improved over time, and a design is referred to as a first, second, third or fourth generation CAR depending on the composition of the costimulatory domains [3]. Effector T-cell functions are independent of MHC recognition since CARs allow T-cell redirection and activation upon the single chain fragment variable (scFv) antigen binding [3]. CAR T-cell therapy has recently shown excellent results in the treatment of hematological malignancies, particularly in patients with refractory and relapsed B-cell acute lymphoid leukemia (B-ALL) [4,5,6,7,8,9,10], non-Hodgkin lymphoma (NHL) [11,12,13], chronic lymphocytic leukemia (CLL) and multiple myeloma (MM) [14,15,16,17,18,19]. These promising results have led to CAR T-cell approval for B-ALL and DLBCL and suggest a potential transfer of this kind of therapy also in other malignancies. In the last five years the field of CAR T-cell therapies has experienced an exponential growth and currently, according to clinicaltrials.gov, there are 530 clinical trials testing CAR T-cell therapies and more than half are directed against leukemia.

Despite the first wave of CAR T-cell therapy success, there are several bottlenecks that need to be addressed in order to unlock the full potential of this therapy. As a matter of fact, most of the CAR T-cell therapies approved or investigated in clinical trials rely on viral vectors, such as retroviral and lentiviral vectors. Generally, retroviral vectors are versatile systems considering the high gene expression capacity and the possibility of using multiple stable packaging cell lines available with vast tropism [20]. Moreover, several long-term follow up studies have demonstrated the safety of the retroviral vectors in the context of adoptive T-cell therapy. Lentiviral vectors are broadly used as they are capable to transduce non-dividing cells, except for cells in G0 phase, and have shown a safer integration profile, particularly relevant for the transduction of hematopoietic stem cells [21,22], together with efficient gene transfer. While lentiviral vectors display those appealing features, the lack of widely available stable vector packaging systems, limitations in the lot size and lot-to-lot variability due to the required multi-plasmid transient transfection procedure [23] make their accessibility complicated and expensive [24,25]. For these reasons, researchers are looking for more affordable gene transfer methods able to combine the favorable attributes of integrating viral vectors (i.e., efficiency in genomic insertion) whilst eliminating or at the least significantly reducing the inadvertent side-effects (i.e., insertional oncogenesis).

Transposable elements (transposons) could potentially offer such an alternative with a vast potential for diverse applications in genetic engineering, including CAR T-cell therapies [26,27,28,29,30]. In this review we will discuss the employment of a non-viral Sleeping Beauty transposon-based CAR-gene transfer approach, which is overall associated with less cumbersome protocols and reduces the cost of goods, as a unique alternative to current viral-based strategies.

## 2. Preclinical Evolution of Sleeping Beauty Vector Design

### 2.1. Transposons as Vectors for Gene Therapy

Transposable elements have been identified as being natural, mobile genetic elements able to change their location within the genome, moving from a donor site to an acceptor site and have played an important role in the evolution and speciation. Transposable elements are subdivided into two classes of transposable elements, the retro-transposons and the DNA transposons, according to the mechanism of transposition. Retro-transposons undergo transposition via an RNA intermediate through a “copy-and-paste” mechanism and include Long Terminal Repeat (LTR) transposons, long interspersed nuclear elements (LINE) and short interspersed nuclear elements (SINE). LINE are the only active mobile elements in humans, while SINE are the most abundant transposable elements in the human genome that might transpose when other elements are introduced, being non-autonomous elements. The DNA-transposable elements, which are the ones used for the non-viral engineering application, move via a DNA intermediate through a “cut-and-paste” mechanism. DNA transposons stably integrate, through a precise recombinase-mediated mechanism, into chromosomes, providing long-term expression of the gene of interest.

Up to date, no vertebrate DNA transposons have been found and their application to vertebrate engineering takes advantage of reconstructed fossil invertebrate transposons derived from fish and insect genomes. In 1997, a versatile transposon became available to be used as an efficient delivery system in mammalian cells. It was engineered from ancient Tc1/mariner transposon fossils found within the Salmonid genomes by in vitro evolution and was named Sleeping Beauty (SB) transposase as a metaphor of the Grimm brothers’ famous fairy tale underlining the concept of awakening towards new applications [26,27,28,29,30]. SB transposase represents the first functionally active enzymatic factor that catalyzes the process of transposition and was constructed based on sequences of transpositionally inactive elements isolated from fish genomes. SB is active in a wide series of vertebrates, including human cells. PiggyBac is another DNA-transposon system derived from the cabbage looper moth *Trichoplusia ni* in the 1980s. It has been employed for genomic modification of mammalian cells from 2005. Since then, piggyBac has become, together with SB transposon, one of the most exploited non-viral gene transfer systems [31].

Both the SB and piggyBac transposon systems consist of two components: The engineered transposon, which carries the gene of interest to be inserted into the genome flanked by inverted terminal repeats (ITRs), and the transposase, which catalyzes the process of “cut-and-paste” transposition. Thanks to a “cut-and-paste” mechanism, mediated by the transposase recognition of the ITR elements, the transposon is mobilized from the plasmid DNA to an acceptor site within the genome. SB transposase inserts transposons into highly abundant TA sequences in the genome [32,33]. PiggyBac transposase inserts transposons in TTAA sequences and was shown to have a higher activity for transposon mobilization than SB in mammalian cells [34]. Single expression unit cassette, as well as multicistronic cassettes including multiple features (i.e., genes and control regions) can be designed. Transposons have a greater genetic payload capacity than viral vectors (up to 8 kb), though the transposition efficiency decreases with increasing insert size. The ideal cargo size for SB transposons is under 6 kb. Still, reasonable transposition rates can be achieved using longer cargos (up to 11 kb), which could be further improved by using a sandwich configuration [35,36]. Other advantages of transposons include their simplicity of use and overall cheaper production costs for clinical implementation. In addition insertional mutagenesis caused by the integration of vector DNA into host cells near an oncogene is a potential concern with all integrating viral vectors, although lentiviral vectors may have a lower risk of mutagenesis [22]. Retroviral vectors have the tendency to target gene transcriptionally control regions as promoters, thereby having an increased probability to induce aberrant gene expression. Conversely, lentiviruses integrate preferentially inside actively expressed genes, potentially leading to interruption of gene expression and potential expression of gene fragments. SB transposon technology shows a close-to-random integration pattern profile without any preference for actively transcribed genes. The lack of bias for integration within transcriptionally active regions of the genome and in regions near the transcriptional start site (TSS) results in a safer genotoxicity profile. The non-viral system is theoretically not pathogenic, even though a more rigorous assessment will be possible as the number of clinical applications increases. Furthermore, being a nucleic acid-based vector, SB transposons have negligible immunogenicity.

In the next sections, we will focus our attention primarily on the SB transposon system for CAR T-cell engineering. The use of transposons for other applications falls outside the scope of this review.

### 2.2. Evolution of the SB Transposon System and Delivery Technologies

Both SB transposon and transposase have been extensively optimized in the last two decades to improve transpositional activity. Approaches including codon optimization of the transposase, the engineering of hyperactive transposases by means of amino acid substitutions and modification of transposon terminal repeats have improved transposition efficiency, enabling stable gene transfer in both stem/progenitor cells and differentiated cell types.

Transposon and transposase can be provided in the same molecule (cis configuration) or in two different molecules (trans configuration). While the cis approach seems to be more straightforward (considering that only a single plasmid needs to be delivered into the cells), the trans configuration has been utilized in most applications [37]. The advantage of the trans approach is the ratio of the transposon: Transposase plasmids can be independently controlled in order to enhance the transposition efficiency. The physical separation of the transposon from the transposase also provides the possibility of supplying the transposase in forms other than DNA, as an mRNA molecule or even as a soluble protein [38,39].

So far, the modification of nucleotide residues (including mutations, deletions and additions) within the ITRs of the original SB transposon (pT) have resulted in improved transposon versions, such as pT2, pT3, pT2B and pT4 [33,40,41]. In parallel, various screens mutagenizing the primary amino acid sequence of the SB transposase have resulted in hyperactive transposase versions; the SB transposase is a 39 kDa protein comprising DNA binding domains, a nuclear localization signal (NLS) and the catalytic domain, featured by a conserved amino acid motif (DDE). The original SB10 transposase could facilitate the integration of a plasmid DNA by 40-fold [26]. Starting from the SB10 transposase, the mutagenized hyperactive versions include SB11 (3-fold higher than SB10), SB12 (4-fold higher than SB10), HSB1–HSB5 (up to-10 fold higher than SB10), HSB13–HSB17 (up to 17-fold higher than SB10), SB100X (100-fold higher than SB10) and SB150X (130-fold higher than SB10) [42,43]. The latest version of the SB system is the use of the hyperactive SB100X transposase in association with the pT4 transposon [33,42,43].

In developing non-viral gene delivery strategies, the most important challenge is to design a delivery system that simultaneously achieves high efficiency of gene delivery, prolonged and stable gene expression and low toxicity. The most used technique to introduce nucleic acids into cultured cells is transfection: DNA is mixed with a carrier molecule (compounds, polymers or cationic lipids) that mediates its entry into the cells also facilitated by an electric or magnetic force. Electroporation is a relatively harsh delivery technology as many cells can die during the process; on the other hand, it is applicable to almost all types of cells, including primary cells. Cells suspended in a solution are exposed to high-voltage pulses of electricity, which creates transient pores in the cell membrane, promoting DNA entry. The nucleofection is an electroporation-based technology whereby the nucleic acids are delivered directly into the cell nucleus and has proved effective in transfecting “hard-to-transfect” cells, such as stem cells, neurons and other primary cells. Many efforts have been made so far to increase the transduction efficiency of transposon systems, which was inferior to viral methods and limited their application. Among the most used commercial devices there are AMAXA Nucleofector (LONZA, Basilea, Switzerland) Neon system (Thermo Fisher Scientific, Waltham, MA USA) and MaxCyte electroporator (Gaithersburg, MD, USA).

### 2.3. CAR T-Cells Engineered With Sleeping Beauty Transposon Vectors

The first attempts in the early 2000s aimed at the genetic manipulation of T-cells to express CAR molecules relied on electrotransfer of plasmid encoding for an anti-CD19 CAR coupled to antibiotic resistance for the subsequent selection and expansion of CAR T-cells. This latter step was necessary since the sole electrotransfer of naked DNA was associated to low rates of transfection [44]. To date, the advent of the SB transposon system in the CAR T-cell engineering field has greatly improved and simplified the non-viral manipulation strategies.

The pioneering works in the US of Dr. Huang at the University of Minnesota, where the SB technology was invented, and of Dr. Cooper at the MD Anderson Cancer Center (MDACC, Houston, TX, USA) in 2008 started to point out all the advantages of using the SB transposon system over the widely used viral methods or the other naked DNA vectors [45,46]. Their studies demonstrated the feasibility of SB technology to generate CD19 CAR T-cells starting from either peripheral blood or cord blood cells. In the study by Huang et al., the relatively low CAR transfection efficiency was overcome by using a CD20 selection marker for the enrichment of CD19 CAR T-cells, while Cooper et al. employed a weekly stimulation step by CD19+, activating and propagating cells (AaPCs) to select for the expansion of anti-CD19 CAR T-cells.

In 2011, the new hyperactive SB100X transposase [43] was compared to SB11 for the generation of CAR T-cells, resulting in 10 to 100 times more efficient in its transpositional activity and 3 to 4 times more efficient in the outgrowth of anti-CD19 CAR T-cells. These results were obtained by reducing by 10 times the amount of SB100X transposase plasmid as compared to SB11 or, most importantly, by using the SB100X mRNA [38]. Indeed, the transient expression of transposase given by the mRNA would reduce the toxicity associated to DNA nucleofection and increase the safety, avoiding potential integration of the transposase within the genome and consequent transposon remobilization [38]. The use of mRNA as transposase source is particularly relevant when applied to SB100X, since SB100X may support multiple integrations compared to single integrations produced by SB11 [43].

The robustness of the SB non-viral approaches for acute leukemia treatment became increasingly evident as other CARs (ROR1 and CD123 CARs) and also other effectors were preclinically tested [47,48]. Thanks to the collaboration with the MD Anderson Cancer Center, our group adopted the SB transposon system for the expression of CD19 and CD123 CARs in a non-conventional T-cell population named Cytokine-Induced Killer (CIK) cells characterized by the enrichment of CD3+ CD56+ cytotoxic cells [49,50]. The safety profile (low GVHD occurrence) and the ease of manufacturing make CIK cells an appealing effector T-cell population to be redirected with CARs [50,51]. We developed an improved platform for SB-mediated engineering by performing a single stimulation step in the presence of OKT3 and *γ*-irradiated autologous PBMCs as feeder. With this platform, we achieved up to 60% CAR expression levels (SB11 and pT) for both the CD19 and CD123 CARs. Notably, CAR-redirected CIK cells were in vitro and in vivo functional against ALL and AML cell lines. Furthermore, the platform is suitable for conventional T-cells, such as OKT3 and beads-activated T-cells [48]. Our method of ex vivo expansion with autologous feeder cells was indeed applied by other groups for anti-CD123 CAR NK cells and also for T-cell engineering with piggyBac transposon to express CD19 CAR [52,53].

In 2016, another important step forward in the bioengineering of SB transposons was achieved by using Minicircles (MCs) plasmids [54], small supercoiled circular DNA vectors that do not contain some conventional sequences of plasmid backbones, such as antibiotic resistance markers or the bacterial origin of replication. CAR T-cells produced through MC vectors were comparable to those produced by lentiviral vector (LV) transduction in terms of cytotoxic activity, cytokine production and proliferation against CD19+ target cells as well as in a CD19+ lymphoma xenograft model [55]. The use of MC vectors improved both the transposition efficiency and the CAR expression (up to 60%) compared to the use of plasmid-encoded SB transposon. This could be due to the relatively close proximity of the SB transposon ends within the MCs, likely contributing to facilitating transposon/transposase complex formation. In addition, the MCs’ smaller size, facilitating their entry through the cell membranes, consistently reduced the toxicity after nucleofection. A major translational advantage that this technology has is the lack of antibiotic resistance genes, which avoids any potential horizontal transfer of antibiotic resistance to host bacteria and unintended integration into the host genome. MC manufacturing requires quite complex steps of recombination and chromatography purifications and current R&D activities are ongoing to establish processes for high quality grade MC production to accelerate both pre-clinical and clinical implementation [56].

One of the most recent advancement in the SB vectorization has been the recombinant production of a highly soluble SB100X transposase protein (hsSB), which has allowed the clearance of the hsSB protein within 24 h after electroporation. The use of hsSB reduced the overall toxicity of the gene manipulation procedure and the period of active transposition as compared to DNA or mRNA based protocols, allowing tighter control of transgenesis with advantages for the safety profile. The integration rate in T-cells after electroporation with hsSB and an MC encoding for a CD19 CAR was between 20 and 30%. In vitro and in vivo specific antileukemic activity against CD19+ target cells was confirmed as well as a safety profile with a vector copy number of < 5 copies/cell and a favorable close-to-random pattern of integration [39]. This strategy enables the generation of transposase-free CAR T-cells within a time window of three or even fewer days. This is an appealing feature that is in line with some current efforts trying to reduce ex vivo manipulation of CAR T-cells to preserve their longevity and fitness, ultimately resulting in a better antitumor activity.

**Figure 1:**
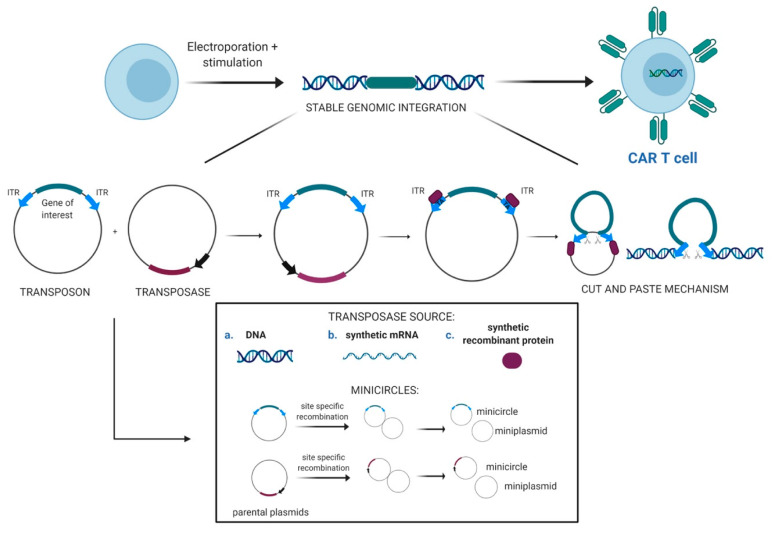
Sleeping Beauty transposon system for CAR T-cell engineering. Two-component DNA transposon-based gene delivery systems: Transposon plasmid carrying the gene of interest flanked by the ITRs and transposase expression plasmid; mechanism of ’cut and paste’ transposition of Sleeping Beauty for a stable genomic integration of the CAR; different transposase sources currently available: plasmid DNA, synthetic mRNA and synthetic recombinant protein; recombination of parental plasmids into minicircles and miniplasmids. ITR, inverted terminal repeat. Created with Biorender.com

Recently, Chicaybam et al. reported on a simplified and clinically applicable protocol for the generation and ex vivo expansion of SB-engineered CD19 CAR T, envisioning 8 days of ex vivo expansion after nucleofection. The resulting cell populations were assayed at the end of the 8-day culture period and were shown to elicit robust antileukemic activity both in vitro and in vivo. The majority of CAR T-cells had a central memory (CM) (CD45RO−CD62L+) phenotype, in both CD8+ and CD4+ subpopulations, thus representing a favorable phenotype to ensure a persistent antitumor effect [57]. Generation of CAR T-cells under a point-of-care approach will allow a more rapid (<2 days) manufacture in absence of ex vivo activation and expansion, further widening the perspectives of simplified, cost effective and more accessible CAR T-cell therapy [58,59]. The common steps of the application of the SB transposon system to CAR T-cell engineering are illustrated in Figure 1.

## 3. Clinical Grade Production of SB-Engineered CAR T-Cells: Cell Processing and Release Testing

Most of the current CAR T-cell trials use patient-derived cells from non-mobilized apheresis and viral vector-mediated engineering. The whole procedure is generally complex and includes several steps such as collection and quality checks of apheresis, CD3+ T-cell selection and enrichment, T-cell activation in vitro, transduction using lentiviral or retroviral vectors, T-cell expansion and cryopreservation of the final product. Each step presents specific issues in the perspective of a more reproducible, safe and scalable production process [60]. Viral vectors are considered as a raw material and require high levels of quality control and stability checks [61,62,63] and their manufacture can take a minimum of 2 weeks. Then a further GMP process, involving sterilizing filtration and filling under aseptic GMP conditions must be performed [64]. The complexity of the application of viral methods for CAR T-cell transduction has a strong impact on the cost of the whole process [65].

**Figure 2:**
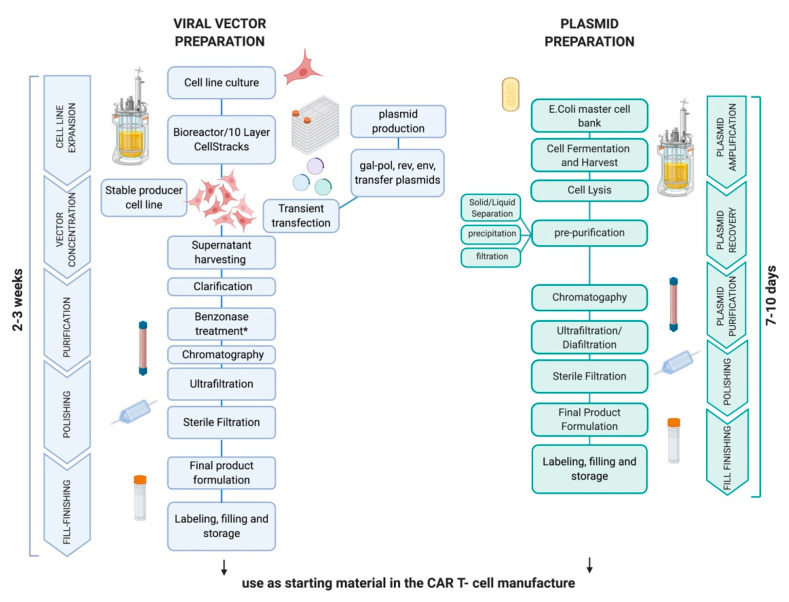
Flow diagram for viral vector and plasmid manufacture. The process of viral production relies on the vector production by stable producer cell lines in the case of retroviral vectors or by transient cell line transfection for lentiviruses. The viral supernatant undergoes concentration, purification and formulation steps. *Benzonase treatment applies only to lentiviral vectors. For plasmid production, initially the E Coli master cell bank of the intended plasmid is produced, from which plasmid replication is promoted by fermentation. After cell lysis, the pre-purification step can be performed by means of filtration, precipitation or solid–liquid separation, followed by the purification and formulation phases. Created with Biorender.com

The production of nucleic acids is possible on a large scale by a multistep process, allowing the production of CAR T-cells for multiple patients with the same batch and therefore a reduction in costs (Figure 2). The non-viral transposon-mediated engineering of CAR T-cells uses either DNA plasmids, MC or RNA, and electroporation as a delivery method. Some of the release criteria associated with the transposon genetic technology are distinct from the common viral vector testing, while others are shared. However, both technologies need to evaluate the vector copy number (VCN) using PCR at the end of the production process. For transposon the specific batch release threshold was assessed for values below around five copies of VCN per single cell. When providing the transposase in DNA format, a safety release test is required to evaluate the number of enzyme molecules in the cells at the end of production process. Clearly, testing for replication competent retrovirus (RCR)/lentivirus (RCL) only applies to viral methods. The most relevant parameters for CAR T-cell batch release are provided in Table 1.

So far CD19-specific CAR+ T-cells have been efficiently expanded after SB electroporation, generating large numbers of GMP-grade CD19-specific CAR T-cells for clinical applications [46,66,67].

Electro-transfer of the SB transposon (CD19RCD28) and the SB transposase (SB11) plasmids was followed by addition of γ-irradiated AaPC in the presence of IL-2 and IL-21 [68,69]. Based on our pre-clinical findings on the use of irradiated autologous PBMCs to overcome cell death following SB-mediated gene transfer, we translated our pre-clinical SB platform in full GMP conditions. The production was performed with the purpose to provide the proof-of-concept for the safety and efficacy of SB-engineered donor-derived anti-CD19 CARCIK cells [50] in relapsed and refractory B-ALL. Specifically, the cellular product was generated starting from 50–60 mL of peripheral blood (PB) by electroporation with the GMP-grade CD19.CAR/pTMNDU3 and pCMV-SB11 SB plasmids according to the method enclosed in the filed patent EP20140192371. The manufacturing process and the quality control tests were performed in a GMP academic cell factory authorized by Agenzia Italiana del Farmaco (AIFA) [70] in the context of an ongoing phase I-II clinical trial (NCT03389035) for children and adults with relapsed/refractory B-cell precursor ALL post-HSCT. The median duration for the production of a single batch was 23 days. All the batches were largely sufficient to treat the enrolled patients with a fold increase in the cell expansion of ≥70 times. CARCIK-CD19 cells were mostly CD3+CD8+ lymphocytes with an average of about 40% of CAR expression. Final products were largely compliant with safety release parameters including the transgene copy number/cells and the SB11 transposase copies in addition to microbiological testing. All the produced 20 batches demonstrated very high cell vitality (≥90%) and were highly cytotoxic in vitro towards CD19+ cell targets. Cell products appeared to be highly polyclonal and no signs of genotoxicity by transposon insertions could be observed by integration site analysis. All the GMP batches were released about 10 days after the end of production. These data demonstrate that our GMP platform is feasible and allows rapid and efficient expansion of highly potent CAR T-cells starting from easily available small amounts of PB [71].

Another important issue in the perspective of CAR T-cell scaling-up is the automation of the cell culture step. Currently, most systems are manually conducted or semi-automated and bioreactors can represent technical solutions potentially able to overcome the concerns of safety and reproducibility in growing large amounts of T-cells. However, most of them still need to be coupled to different devices for the sequential steps of the production process [72,73,74,75,76]. A fully automated closed system, the CliniMACS prodigy (Miltenyi Biotec, Germany), has been recently proposed and validated for lentiviral transduced CAR T-cells in several clinical experimental settings [77,78,79].

## 4. Early Clinical Experiences

Transposon engineering for clinical application has been mostly exploited pre-clinically (reviewed in Hodge et al. 2017 [80]) and barely a few pilot clinical experiences have been activated so far, as summarized in Table 2. The first clinical application of transposon-mediated gene therapy was explored by the pioneer work at MDACC with encouraging results [30]. In this study, T-cells were genetically modified using pT2 and SB11 DNA plasmids to stably express a second-generation CD19-specific CAR containing a CD28 costimulatory molecule, and selectively propagated ex vivo with multiple stimulations by using AaPCs and cytokines. Twenty-six patients, both adult and children among them, with advanced non-Hodgkin lymphoma and B-ALL safely underwent HSCT and infusion of CAR T-cells as adjuvant therapy in the absence of lymphodepletion in the autologous (*n* = 7) or allogeneic settings (*n* = 19). SB-mediated genetic transposition and stimulation resulted in 2200- to 2500-fold ex vivo expansion of genetically modified T-cells, with 84% CAR expression, and without integration hotspots. No acute or late toxicities and no exacerbation of GVHD were observed. Absence of B-cell aplasia was associated with limited in vivo expansion, which occurred in the absence of lymphodepletion and low tumor burden at the time of CAR T-cell infusion. Nevertheless, CAR T-cells were detected by transgene PCR up to an average of 201 and 51 days for autologous and allogeneic recipients, respectively. More recent evidence, within the scope of a long-term follow-up study, reveals that four out of seven patients had persistent CAR T-cells detected after a median period of 4.7 years [81]. Overall, those data suggest that SB engineering is feasible, safe and allows stable engraftment of modified T-cells with efficiencies comparable to viral vectors.

The major drawbacks of this first-generation SB-engineered CAR T-cells are the multiple stimulations provided by the co-culture of T-cells with irradiated AaPCS (K-562 cell line modified to co-express CD19 and co-stimulatory molecules), requiring a 4-week manufacturing process. To solve this issue, MDACC has exploited a second-generation approach which reduced T-cell manufacture on feeder cells to two weeks and applied a revised stalk derived from CD8 [82]. By using this platform, autologous CAR T-cells have been infused combined with Fludarabine and Cytoxan lymphodepletion in adult and pediatric patients with active CD19+ diseases (NCT02529813). This approach is part of a multi-step planning by Ziopharm (Boston, MA, USA) and MD Anderson. The emerging results will be the basis for providing data to support third-generation trials using the previously described 2-day manufacture platform for CAR+ mbIL15+ Switch T-cell generation [58]. In collaboration with the National Cancer Institute (NCI) and Precigen under a cooperative and development agreement (CRADA), Ziopharm is expanding the application of this concept of T-cell electroporation and consecutive infusion in the patient without pre-activation. A phase II trial using autologous neoantigen-specific T-cell receptor (TCR) engineered T-cells is currently recruiting patients with metastatic solid cancers (NCT04102436).

**Figure 3:**
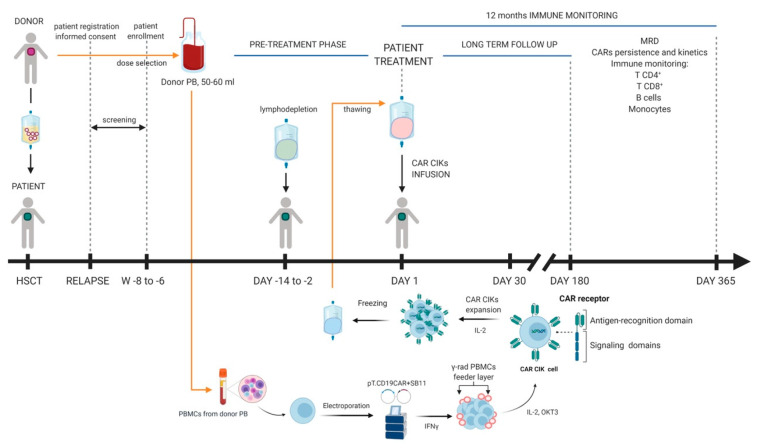
Overview of donor-derived SB-engineered CARCIK-CD19 therapy; 50–60mL of donor PB are collected and PBMCs are isolated and nucleofected at day 0 with SB vectors. Gamma-irradiated PBMCs are added as feeder together with IFN-*γ*; at day 1 cells are stimulated with IL-2 and OKT3 and CARCIK-CD19 cells are expanded in vitro and frozen on days 18–21; during the pre-treatment phase (days 2 to 14) the patient undergoes lymphodepletion and then, on day 1 of the treatment phase, CARCIK-CD19 are thawed and subsequently infused into the patient; after the infusion, the patient is subjected to a long-term follow-up until the fifth year and to 12 months immune monitoring. Created with Biorender.com

We are currently running a multicentric phase I/II clinical trial to treat patients with B-ALL relapsed post allo-HSCT at the MBBM pediatric unit in Monza and at the ASST Papa Giovanni XXIII adult unit in Bergamo (NCT03389035). The design of the trial is based on the results from a pre-clinical study and follows a dose-escalation scheme [85]. After standard lymphodepletion, patients are treated with a single infusion of CAR T-cells, generated by SB non-viral manipulation of PBMCs freshly isolated from 50 mL of blood of the previous transplant donor (Figure 3). The SB plasmids employed are the SB11 transposase and pT transposon expressing a CD19 third generation CAR incorporating the CD28 and OX40 costimulatory domains. To limit GVHD, we opted for T-cells differentiated ex vivo by using the CIK cell protocol and we named the cellular product CARCIK-CD19. Notably, sequential infusions of unmanipulated donor lymphocytes and CIK cells after allo-HSCT have been previously adopted by our centers (NCT01186809) [86]. Among the 74 patients, including 16 children and 58 adults, enrolled into the study (median age 45, range 1–67), 20 had a diagnosis of ALL (27%) and 41 of AML (55%). All patients relapsed after matched allogeneic transplants (32 unrelated and 42 sibling), of whom 44 (59%) suffered from a hematological, 4 (5%) from a cytogenetic and 26 (35%) from a molecular relapse. Non relevant acute organ toxicity was shown, with no incidence difference among adult and pediatric patients. As per protocol, clinical response was determined 100 days after the last unmanipulated CIK administration and the study was analyzed on an “intent to treat” basis. An overall response rate of 36% was observed. Acute GVHD was observed in a total of 11 patients (15%): grade 1 (*n* = 4), 2 (*n* = 2) and 3–4 (*n* = 5). During follow up, chronic GVHD was observed in 8 patients (11 %) (3 mild, 4 moderate and 1 severe). Those results clearly demonstrate the safety and tolerability of repeated CIK cell infusions with minimal and manageable GVHD occurrence, providing the rationale of using donor-derived CAR T-cells over conventional autologous CAR T-cells in our trial [87]. We strongly believe that this approach has potential advantages in terms of cell availability and sustainability, when applied to a patient population failing autologous T-cell manufacture.

So far, we have treated 4 pediatric and 9 adult patients. All the patients had a history of multiple prior lines of therapy and at least one allo-HSCT. The cellular product was derived from HLA identical sibling, matched unrelated donor (MUD) and even haploidentical donor. Preliminary results indicated robust expansion and persistence of SB-engineered CAR T-cells in the majority of treated patients. Expansion was associated with clear observations of engrafted CAR T-cell functionality and persistent B cell aplasia in the absence of life-threatening toxicities. No evidence of GvHD was found in any of the treated patients and integration site analysis of the patients’ peripheral blood confirmed the unique safety of this engineering approach in terms of genotoxicity [71]. Given that our findings are based on a limited number of patients and short-term follow-up, these results should therefore be interpreted as suggestive rather than definitive. Further data collection is underway and will shed light on the impact of using non-viral approaches for CAR T-cell generation. Nevertheless, these data offer considerable clinical evidence with important implications for non-viral technology coupled to allogeneic product manufacturing.

Recently, the SB transposon system was investigated in clinic for the biodelivery application. SB100X was used to improve nerve growth factor (NGF) secretion by encapsulated cell biodelivery device in patients with mild to moderate Alzheimer’s disease [84]. The use of SB to engineered cells as the biodelivery system will similarly be exploited in a recently activated phase 1/2 clinical trial at the Masonic Cancer Center, University of Minnesota (Minneapolis, MN, USA) for patients with Hurler Syndrome for delivery of alpha-L-iduronidase (IDUA) sponsored by Immusoft (Seattle, WA, USA). In the next few years, an increasing number of clinical studies using SB100X that are currently being proposed and developed will be activated. TargetAMD is a consortium founded by the European Commission coordinated by the University of Geneve for the transposon-based ex vivo gene therapy of age-related macular degeneration (AMD). More details on this topic, which are beyond the scope of this review, can be found in [80]. The use of SB100X in mRNA format and a transposon as MC to engineer CAR T-cells targeting the myeloma antigen SLAMF7 [88] will be investigated in the context of a European multicenter clinical trial [89]. In collaboration with the Max-Delbrück Center for Molecular Medicine (MDC, Berlin, Germany), we are currently exploring the use of the hyperactive SB100X transposase and pT4 transposon for the generation of anti-CD33 CARCIK cells (Rotiroti MC et al., in press, Molecular Therapy 2020 [90]).

In parallel to the SB system, eight patients with r/r CD19+ malignancies post HLA-matched sibling HSCT have been treated with donor-derived anti-CD19 CAR T-cells genetically modified using piggyBac. Anti-CD19 CAR T-cells were produced from low numbers of PBMCs, with CD19 stimulation and IL-15 support [91]. Early results suggest expansion and activity of piggyBac engineered CAR T-cells in a first in-human study [92]. Two additional trials of stem cell memory (Tscm) CAR T engineered with PB are ongoing in the US. A multicentric phase I-II trial, sponsored by Poseida Therapeutics (San Diego, CA, USA) and the California Institute for Regenerative Medicine (CIRM) is conducting a study targeting BCMA for r/r multiple myeloma. The investigators reported durable responses in the absence of important toxicities [93]. A phase 1 anti-PSMA CAR T-cell trial for adult patients affected by metastatic prostate cancer is also ongoing at the City of Hope Comprehensive Cancer Center (Duarte, CA, USA) and the Sarah Cannon Research Institute at HealthOne (Toronto, ON, Canada). Additional phase I trials using piggyBac engineered anti-CD19 CAR T-cells include those at Nagoya University Graduate School of Medicine (Japan) or at Kunming Yan’an Hospital (China). No data are currently available from these trials.

## 5. Future Perspectives of Non-Viral CAR T Platforms

CAR T-cells immunotherapy has indubitably revolutionized cancer treatment. As a breakthrough therapy, new routes of investigation have opened and raised advanced knowledge in the way cancer cells behave and persist upon standard treatments. On the other hand, there are still several issues that need to be further addressed to make CAR T-cell therapy available for different tumor entities and for a higher number of patients [24].

The recent improvements obtained in non-viral transposon-mediated gene transfer approaches for CAR T-cells grant easier and cost-effective manufacturing processes together with a safer integration profile [65]. The SB transposon has been one of the most employed non-viral systems for CAR-engineering, showing comparable efficiency as viral vectors and offering a valid alternative for the gene transfer of both autologous and allogeneic starting cell sources (i.e., cord blood) [45,46]. In view of the shift of the “one batch one patient” paradigm (autologous products) towards “off-the-shelf” products [94], SB transposons satisfy all requirements for easier and more affordable CAR T-cell therapy. In this regard, gene editing such as CRISPR-CAS9 for the generation of universal CARs has started to be coupled to transposons, as a cutting-edge perspective that will surely further facilitate the current available genetic manipulation strategies [95].

Moreover, the identification of target antigens, beyond CD19, that confer efficient and safe antitumor activity represents an active field of research in both hematological and solid contexts. The application of CAR T-cells to solid tumors and some blood cancers such as CLL and AML posed additional challenges related to the hostile immunosuppressive microenvironment, continuously raising the bar toward a more tuned and personalized approach. Future work will concentrate on safely tuning the efficacy of CAR T-cells against tumor cells, whilst avoiding on-target off-tumor toxicities, and on limiting the emergence of tumor escape variants from selective antigen pressure given by single CAR approaches. Responses to these new challenges will be provided by means of smarter CARs, entailing double targeting approaches (dual CARs) and fourth generation armored CARs designed to secrete cytokines, together with combinatorial strategies with other immunomodulatory agents (i.e., checkpoint inhibitors) [96,97]. In this perspective, non-viral transposon procedures can benefit from their increased DNA carrying capacity.

Over the next few years we will likely see the results of these pioneering efforts, which will ultimately aid researchers to further evaluate the feasibility of non-viral gene transfer methods. The questions that need to be answered are whether cells engineered with transposition will be as effective as those virally transduced, and safe in terms of insertional mutagenesis and transposon remobilization.

## Figures and Tables

**Table 1:** Quality control parameters for the release of CAR T-cell products.

Significance	Test	Methods
Quality	Cell Viability	Trypan blue dye exclusion; Flow cytometry
Purity/identity	%CD3+ cells	Flow cytometry
Identity	%CD3+/CAR+ cells	Flow cytometry
Potency	Cytotoxicity/Cytokine production toward target cell lines	Flow cytometry/ detection of cytokines
Safety	Mycoplasma	Culture assay/PCR assay
Safety	Bacterial sterility (aerobic, anaerobic and fungal testing)	BacT/ALERT 3D
Safety/purity	Endotoxin	Different methods
Purity	Contamination of beads, cytokines, serum, etc	Different methods
Safety	Vector Copy number/cell	PCR
Safety	Transposase detection (only for transposon-based CAR T-cells)	PCR
Safety	Replication competent retroviruses/lentiviruses (RCRs/RCLs) (only for viral-based CAR T-cells)	PCR

**Table 2:** Active, recruiting and completed clinical trials using transposon-mediated gene transfer, May 2020.

Disease	Clinical Trial ID/Alias	Location	Population Studied; Phase	Transgene	Vector	Status	Reference
B-cell lymphoma	NCT00968760	MDACC ^8^ (Texas, USA)	Adult; Phase 1	Autologous anti-CD19.CD28.z CAR T-cells	SB	Active, not recruiting	[30]
CD19+ lymphoma, B-ALL ^1^	NCT01497184	MDACC	Children and adult; Phase 1	Allogeneic anti-CD19.CD28.z CAR T-cells	SB	Active, not recruiting	[30]
CD19+ lymphoma,B-ALL	NCT01492036	MDACC	Children and adult; Phase 1	Long-term follow-up	SB	Recruiting	[81]
B-CLL ^2^	NCT01653717	MDACC	Adult; Phase 1	Allogeneic anti-CD19.CD28.z CAR T-cells	SB	Completed	
CD19+ lymphoma, B-ALL, B-CLL	NCT02529813	MDACC	Children and adult; Phase 1	Autologous anti-CD19.CD28.z CAR T-cells	SB	Active, not recruiting	[82]
B-ALL	NCT03389035	MBBM ^9^/PGXXIII ^10^	Children and adult; Phase 1 and 2	Allogeneic anti-CD19.CD28.OX40.z CAR T-cells	SB	Recruiting	[71]
B-ALL, B-cell lymphoma, B-CLL	NCT03579888	MDACC	Adult; Phase 1	CD19.CD8.CD28.CD3.zCAR-mbIL15-HER1t T-cells	SB	Not yet recruiting	[58]
GBM ^3^, NSCLC ^4^, Breast Cancer, GI ^5^/GU ^6^, Ovarian Cancer	NCT04102436	NCI ^11^ (Maryland, USA)	Adult; Phase 2	Autologous neoantigen-specific TCR T-cells	SB	Recruiting	[83]
Alzheimer’s Disease	NCT01163825	KU ^12^ (Sweden)	Adult; Phase 1	Encapsulated Cell Biodelivery of Nerve Growth Factor	SB	Unknown	[84]
MPS IH ^7^	NCT04284254	MCC ^13^ (USA)	Adult; Phase 1 and 2	Autologous IDUA plasmablasts	SB	Not yet recruiting	
B-ALL, B-cell lymphoma, B-CLL	The CARTELL Study	WH, WHC, SCH ^14^ (Australia)	Children and adult; Phase 1	Allogeneic anti-CD19 CAR T-cells	PB ^17^	Recruiting	
B-ALL	UMIN000030984	NUG ^15^ (Japan)	Children and adult; Phase 1	Autologous anti-CD19 CAR T-cells	PB	Recruiting	
B-ALL, B-cell lymphoma	NCT04289220	YAHKMU ^16^ (China)	Adult; Phase 1	anti-CD19.CD28.41BB.z CAR T-cells	PB	Not yet recruiting	

^1^ B-ALL: B-Acute lymphoblastic leukemia; ^2^ B-CLL: B-Chronic lymphoblastic leukemia; ^3^ GBM: Glioblastoma; ^4^ NSCLC: Non-Small Cell Lung Cancer; ^5^ GI: Gastrointestinal Cancer; ^6^ GU: Genitourinary Cancer; ^7^ MPS IH: Mucopolysaccharidosis Type IH (MPS IH, Hurler Syndrome); ^8^ MDACC: MD Anderson Cancer Center; ^9^ MBBM: Fondazione MBBM; ^10^ PGXXIII: ASST Papa Giovanni XXIII; ^11^ NCI: National Cancer Institute; ^12^ KU: Karolinska University Hospital; ^13^ MCC: Masonic Cancer Center, University of Minnesota; ^14^ WH, WHC, SCH: Westmead Hospital, Westmead Children’s Hospital, Sydney Children’s Hospital; ^15^ NUG: Nagoya University Graduate School of Medicine; ^16^ YAHKMU: Yan’an Affiliated Hospital of Kunming Medical University; ^17^ PB: piggyBac.
